# Endocrine Significance of SARS-CoV-2’s Reliance on ACE2

**DOI:** 10.1210/endocr/bqaa108

**Published:** 2020-07-11

**Authors:** Eric Lazartigues, Mirza Muhammad Fahd Qadir, Franck Mauvais-Jarvis

**Affiliations:** 1 Department of Pharmacology & Experimental Therapeutics, New Orleans, Louisiana; 2 Cardiovascular Center of Excellence, Louisiana State University Health Sciences Center, New Orleans, Louisiana; 3 Southeast Louisiana Veterans Health Care Systems, New Orleans, Louisiana; 4 Division of Endocrinology & Metabolism, Department of Medicine, Tulane University School of Medicine, New Orleans, Louisiana

**Keywords:** renin-angiotensin system, ACE2, TMPRSS-2, SARS-CoV-2, COVID-19, diabetes

## Abstract

The current COVID-19 pandemic is the most disruptive event in the past 50 years, with a global impact on health care and world economies. It is caused by severe acute respiratory syndrome coronavirus-2 (SARS-CoV-2), a coronavirus that uses angiotensin-converting enzyme 2 (ACE2) as an entry point to the cells. ACE2 is a transmembrane carboxypeptidase and member of the renin-angiotensin system. This mini-review summarizes the main findings regarding ACE2 expression and function in endocrine tissues. We discuss rapidly evolving knowledge on the potential role of ACE2 and SARS coronaviruses in endocrinology and the development of diabetes mellitus, hypogonadism, and pituitary and thyroid diseases.

Angiotensin-converting enzyme type 2 (ACE2 [EC 3.4.17.23]), was originally discovered in 2000 as a monocarboxypeptidase located on the cell surface and capable of cleaving the potent vasoconstrictor angiotensin (Ang)-II to the vasodilatory heptapeptide Ang-(1-7) (1, 2). Beyond the renin-angiotensin system (RAS), ACE2 is also able to cleave other peptides such as apelin-13, apelin-36, des-Arg^9^-bradykinin, β-casomorphin, neocasomorphin, and dynorphin (3). As researchers started learning about ACE2, it was unexpectedly identified (4) as an entry point for the severe acute respiratory syndrome coronavirus (SARS-CoV) that spread through China and other countries in 2002-2003. With the SARS epidemic declining over time, research related to ACE2 and the coronavirus soon subsided and the focus shifted back to the cardiovascular field and endocrine role of the enzyme. ACE2 has been shown to play a pivotal role in the “compensatory axis” of the RAS (ACE2/Ang-(1-7)/Mas) (5), opposing the effects of the classical axis (ACE/Ang-II/AT_1_ receptor), with implications for blood pressure regulation (6-9), heart failure (10-12), diabetes (13-16), and obesity (17, 18).

In late 2019, ACE2 was again identified as a receptor for another coronavirus, SARS-CoV-2, responsible for the COVID-19 pandemic (19). As of June 22, 2020, at least 9 million individuals have been infected and 470 000 have died worldwide. Importantly, clinical reports suggest that preexisting conditions such as hypertension, diabetes, and obesity predispose to COVID-19 mortality (20, 21). Here, we review the role of ACE2 as a SARS-CoV-2 receptor as it relates to endocrine diseases.

## ACE2 as a SARS-CoV-2 Receptor

**Figure 1.**
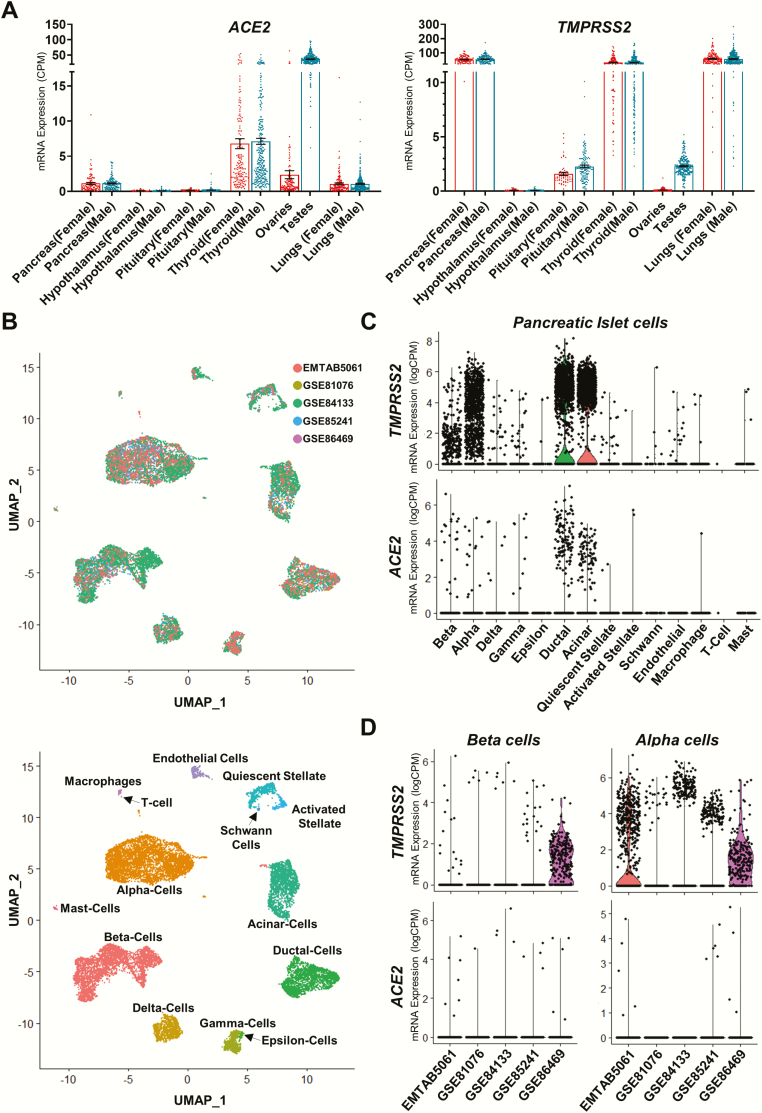
**Patterns of gene expression for ACE2 and TMPRSS2 across select human tissues and pancreatic islet cells.** (A) Scatter plots showing mRNA expression in various human male and female tissues. Left panel: ACE2 expression levels; right panel: TMPRSS2 expression levels. Data shown are derived from the human protein atlas and genotype tissue expression project (26-28). (dbGap accession number: phs000424.vN.pN expression data accessed on May 30, 2020.) n = 34-293. Data plotted as mean ± SEM. (B) Top panel: Nonlinear multidimensional projection plotting using uniform maximal approximation projection (UMAP) plots for 5 prominent pancreatic islet single-cell RNAseq datasets. Data integration performed using Seurat’s SCTransform function (29)). Bottom panel: UMAP plot showing cellular subtype distribution of human pancreatic islet cells. Each point in both plots represents the relative transcriptional identity of a single cell. (C) Violin plots showing single cell RNAseq data for mRNA expression across all islet cell types. Data are derived from 4 single-cell RNAseq datasets of human pancreatic islets. Top panel: TMPRSS2 mRNA expression levels; bottom panel: ACE2 mRNA expression levels. n = 31. Shape of violin plots shows the probability density of mRNA expression for each cell type, whereas each point denotes the expression of an individual cell. (D) Violin plots showing single-cell RNAseq data for mRNA expression across human pancreatic islet beta cells (left) and alpha cells (right). Top panels: TMPRSS2 mRNA expression levels; bottom panel: ACE2 mRNA expression levels. GSE81076 (30): n = 5; GSE85241 (31): n = 4; GSE86469 (32): n = 8; E-MTAB-5061 (33): n = 10; GSE84133 (34) n = 4 (total cells across 5 datasets = 14 890). Shape of violin plots shows the probability density of mRNA expression for each cell type, whereas each point denotes the expression of an individual cell. (Data for B-D were accessed on May 28, 2020, and analyzed using the Seurat scRNAseq analysis package (35, 36)). ACE2, angiotensin-converting enzyme 2.

Recent work has shown that ACE2, previously identified as an entry point for SARS-CoV (22), is also the main receptor for SARS-CoV-2 (23). The virus’ entry into cells first relies on binding to ACE2 followed by priming of the viral spike protein, mainly by the serine protease TMPRSS2, which is necessary for the viral RNA to enter the cell. TMPRSS2 plays a critical role not only for SARS-CoV-2 infection but also for infection by other coronaviruses and influenza (24). Importantly, for the infection to occur, the target cell must have both ACE2 and TMPRSS2 in proximity. Interestingly, TMPRSS2 is an androgen-regulated gene predominantly expressed in the prostate and with lower levels of expression in the lung, colon, liver, kidneys, and pancreas. Although most of TMPRSS2 is membrane-bound, it has also been identified in extracellular vesicles (25), suggesting that the protease can reach out tissues beyond its expression sites. Overall, ACE2 and TMPRSS2 mRNA are expressed in the same organs in males and females (Fig. 1), suggesting that they could potentially contribute to viral infection of these organs.

## ACE2 Expression and Activity in Endocrine Tissues

In animals and humans including hypothalamus, pituitary, thyroid, gonads, and pancreatic islets, ACE2 expression has been reported in most tissues, including those involved in endocrine functions (Fig. 1). Several groups have examined the implications of ACE2 deletion in murine endocrine organs and the overall beneficial effects of ACE2 gene therapy. Our group was the first to report ACE2 protein expression in the mouse brain, notably in hypothalamic regions associated with food intake and metabolic regulation (37). Mice exposed perinatally to a hypercaloric diet exhibited hypomethylation of the *Ace2* gene, consistent with enhanced ACE2 activity in the hypothalamus (38). We also observed that mice lacking ACE2 exhibited a lean phenotype, whereas, in contrast, transgenic mice expressing human ACE2 selectively in neurons showed increased food intake and body weight, associated with fasting hyperglycemia and glucose intolerance. Interestingly, these metabolic abnormalities coexisted with a reduction in hypothalamic apelin levels, a peptide involved in the regulation of water and food intake, and could be partially normalized following intracerebroventricular infusion of apelin-13 (39). Hypothalamic expression of ACE2 was also found to colocalize with vasopressin, oxytocin, and GABAergic neurons, the latter being critical to the maintenance of an inhibitory input to presympathetic neurons projecting to the kidney (40), supporting a compensatory mechanism to the development of hypertension. In the periphery, we also observed ACE2 expression in the exocrine and endocrine pancreas of the mouse, with location in islets of Langerhans mostly restricted to glucagon-producing α-cells (13, 16, 41), a finding also observed in human islets (Fig. 1) (42). However, a different pattern of expression was observed in rats, with colocalization to β-cells and somatostatin-producing δ-cells (43), which suggests that expression could be species-specific. In the mouse, lack of ACE2 was associated with impaired first-phase insulin secretion and glucose tolerance in both males and females (14, 44) as well as decreased β-cell mass resulting from impaired β-cell proliferation (45). In diabetic mouse models (13, 46), ACE2 expression and activity were reduced in the pancreas, whereas ACE2 gene therapy via injection into the pancreas improved fasting glycemia and glucose tolerance, increased β-cell proliferation, reduced β-cell apoptosis, and increased islet insulin content, without affecting insulin sensitivity (13). Although a possible role for Ang-(1-7) has been suggested to mediate the paracrine effects of ACE2 (13, 42), the detailed mechanism remains to be elucidated. Nevertheless, ACE2 appears to be critical to normal β-cell function and glucose homeostasis.

ACE2 is expressed in the adipose tissue where it is thought to regulate local levels of Ang-II (17). Exposing mice to a high-fat diet was shown to exacerbate angiotensinogen expression and increase ACE2 mRNA possibly via peroxisome proliferator-activated receptor γ activation (17). However, in addition to a rise in blood pressure, the increase in Ang-II was associated with enhanced ADAM17-mediated ACE2 shedding from the cell surface, leading to a reduction in ACE2 activity on adipocytes in male (17) but not in female mice (18). Although deletion of ACE2 selectively in mouse adipocytes did not prevent the development of obesity in females, it resulted in a hypertensive phenotype (47). Similarly, administration of 17β-estradiol to ovariectomized female mice was able to reduce obesity and hypertension in an ACE2-dependent manner (48). ACE2 deficiency in mouse bone marrow cells was also reported to promote an inflammatory phenotype in the adipose tissue (49) and the antiobesity effects of ACE2 have recently been suggested to be mediated by a browning of white adipose tissue (50).

ACE2 is expressed in the Leydig cells of the rat testis and in Leydig and Sertoli cells of the human testis with an upregulation occurring at puberty (51). Although these data suggest a role for ACE2 in testicular function, it was independent of the hypothalamo-pituitary-testicular axis.

In summary, these studies demonstrate that ACE2 plays a critical role in metabolic and endocrine regulation in rodents by alleviating overactivity of the classical RAS axis.

## ACE2, TMPRSS2, and Sex Differences in COVID-19

Men are more frequently hospitalized, develop more severe complications, and are 1.5 to 2 times more likely to die from COVID-19 than women (52-57). Notably, ACE2 is located on the X chromosome (58) and human ACE2 promoter activity was also shown to be downregulated by chromosome Y genes, SRY, and Sox3 in testis (59). Because women have 2 copies compared with 1 in men, ACE2 may be regulated differently in men than in women. Yet, current studies suggest that ACE2 expression is not different between males and females (Fig. 1) and between younger or older subjects (60). Therefore, ACE2 might not be responsible for the apparent gender differences in COVID-19 infection. Interestingly, TMPRSS2 is a direct androgen receptor target gene and its expression is increased by androgens in prostate cancer (61), which could also have an impact on TMPRSS2 expression in men with COVID-19. Indeed, an Italian study reported that patients with prostate cancer receiving androgen depletion therapy had a significantly 4-fold lower risk of SARS-CoV-2 infection compared with patients who did not undergo this treatment (62). Although a differential regulation of ACE2 and TMPRSS2 in men and women may affect virus entry and pathogenicity, studies are needed to address these questions.

## ACE2, SARS-CoV-2 Infection, and Pancreatic Islet Dysfunction

SARS-CoV-2 may also affect the endocrine and exocrine pancreas as ACE2 and TMPRSS2 are expressed across the entire spectrum of pancreatic endocrine and exocrine cells (Fig. 1). In islet cells ACE2 and TMPRSS2 mRNA expression appears to be highest in subsets of beta and alpha cells. Exocrine, ductal, and acinar cells also express ACE2 and TMPRSS2 mRNA (Fig. 1). In past decades, 2 other coronavirus outbreaks have crossed species and used the machinery of the endocrine system as a port of entry into cells. The first deadly coronavirus of 2002 (SARS-CoV) also used ACE2 (23). Because SARS-CoV-2 is closely related to SARS-CoV, knowledge accumulated from SARS can be useful to predict what may happen in patients who recover from COVID-19. In the mouse, we and others showed that ACE2 protein is expressed in the islet glucagon-producing α-cells (16). Notably, human islets of Langerhans also express ACE2 protein in α-cells and a subset of other non-ß-cells (42) and the acute onset of new diabetes was reported in 20 patients hospitalized for SARS-CoV and in the absence of glucocorticoid treatment (63). Hyperglycemia was reversible after a few months in most patients, suggesting that SARS-CoV may have entered islet cells using ACE2 as its receptor and produced transient islet dysfunction leading to acute diabetes (63). More recently, in a series of 52 patients hospitalized in Wuhan for COVID-19 pneumonia, 9 (17%) exhibited pancreatic injury with elevated amylase and lipase (64). Among those, 6 patients developed hyperglycemia. Pancreatic injury in COVID-19 subjects may be caused by the direct cytopathic effect of SARS-CoV-2 in endocrine and exocrine cells. However, pancreatic injury could also be the consequence of the systemic responses to respiratory or multiorgan failure or the exaggerated immunoinflammatory response induced by SARS-CoV-2 infection. Viruses have been implicated in the development of type 1 diabetes, and SARS-CoV-2 could be an environmental trigger. Apart from direct β-cell damage, generation of new self-antigens and subsequent immune-mediated destruction of β-cells could be implicated. In addition, infection of the surrounding exocrine pancreas by SARS-CoV-2 may produce islet cell dysfunction via release of inflammatory mediators. Studies are needed to determine the potential effect of SARS-CoV-2 in islet dysfunction and its relation to diabetes pathogenesis.

## ACE2, SARS-CoV-2 Infection, and Testicular Dysfunction

Testes are one of the original tissues where ACE2 expression was first described (1) and were found to be one of the sites with highest ACE2 expression in 3 independent RNA expression databases (Human Protein Atlas, FAMTOM5, and GETx), consistent with prior reports (65, 66). It is also a site of TMPRSS2 expression (Fig. 1). Viruses such as HIV, hepatitis B, and mumps can cause orchitis and, in some cases, male infertility. The analysis of autopsy specimens of testes from 6 patients who died of SARS in Beijing, China, showed testicular involvement with orchitis (66). Testes from the SARS cases exhibited extensive germ cell destruction, with few or no spermatozoon in the seminiferous tubules, as well as macrophage and lymphocyte infiltration compared with non-SARS specimens of testis. Although ACE2 is highly expressed in human testis, SARS-CoV RNA was not detected in the specimens, arguing for an immune-mediated orchitis rather than a direct effect of the virus (66). Testosterone levels in patients with COVID-19 are not a reliable marker of testicular function as the acute and severe infection can suppress the hypothalamic-pituitary-testicular axis and decrease circulating testosterone. However, an unpublished study in COVID-19 patients found that although testosterone levels did not statistically decrease in the COVID-19 group, a significant increase in serum LH concentrations and a decrease in serum ratio of testosterone:LH were observed (67). This suggests that SARS-CoV-2 infection produces Leydig cell failure to produce enough testosterone leading to hypothalamic compensation. Whether this is a direct toxic effect of SARS-CoV-2 or secondary to systemic disease remains to be established. Therefore, monitoring of gonadal function seems warranted in men that recover from COVID-19 as a potential new cause of sterility.

## ACE2, SARS-CoV-2 Infection, and Pituitary and Thyroid Dysfunction

Other endocrine complications of the SARS-CoV infection have been described. The most frequent endocrine complication in a series of 61 patients assessed 3 months after recovery from SARS in Singapore was a transient hypothalamic-pituitary-adrenal (HPA) axis dysfunction in 39% of patients leading to hypocortisolism, which resolved within a year (68). SARS was also complicated by cases of hypothalamic-pituitary-thyroid axis dysfunction with central hypothyroidism (5%). Most cases were reversible (68), and it has been suggested that this post-SARS sickness syndrome is a sudden reversal of a state of chronic cortisol hypersecretion (secondary to infection), leading to a transient condition characterized by decreased HPA axis activity (69). Autopsies of fatal cases of SARS have also revealed evidence of primary injury of the thyroid with apoptosis of follicular cells (70). Thus, monitoring of HPA and thyroid function will likely be justified in the first year following recovery from COVID-19. In fact, ACE2 and TMPRSS2 exhibit high mRNA expression in the thyroid in both sexes (Fig. 1); the first case of subacute thyroiditis (a thyroid dysfunction due to a viral infection or a postviral inflammation of the thyroid) associated with SARS-CoV-2 infection was reported in a young Italian woman (71).

## Conclusions

Altogether, preclinical studies suggest that ACE2 plays a critical role in metabolic and endocrine regulation by preventing RAS overactivity. Although the role of ACE2 in hormonal regulation, notably within the hypothalamus-pituitary axis, is unclear, the enzyme tends to protect against obesity, diabetes, and hypertension.

With respect to COVID-19, clinical studies show more severe outcomes in patients with diabetes, obesity, and hypertension. However, the lack of data in humans on ACE2 expression in pathological conditions in endocrine tissues does not allow us to conclude on a direct role of ACE2 expression in severe COVID-19 outcomes. Data are needed in patients to determine whether these comorbidities are associated with enhanced or reduced expression of ACE2, or TMPRSS2, that could affect SARS-CoV-2 infection.

## Abbreviations

ACE2: angiotensin-converting enzyme type 2
Ang-II: angiotensin II
CoV: coronavirus
HPA: hypothalamic-pituitary-adrenal
RAS: renin-angiotensin system
SARS: severe acute respiratory syndrome

## Additional Information

***Disclosure Summary:*** The authors have no conflicts of interest to disclose.

***Data Availability:*** All data generated or analyzed during this study are included in this published article or in the data repositories listed in References.
